# *Wise* Regulates Bone Deposition through Genetic Interactions with *Lrp5*

**DOI:** 10.1371/journal.pone.0096257

**Published:** 2014-05-01

**Authors:** Debra L. Ellies, Androulla Economou, Beth Viviano, Jean-Philippe Rey, Stephenie Paine-Saunders, Robb Krumlauf, Scott Saunders

**Affiliations:** 1 Stowers Institute for Medical Research, Kansas City, Missouri, United States of America; 2 National Institute for Medical Research, The Ridgeway, Mill Hill, London, United Kingdom; 3 Department of Pediatrics, Washington University Medical School, Saint Louis, Missouri, United States of America; 4 Department of Anatomy and Cell Biology, Kansas University Medical School, Kansas City, Kansas, United States of America; 5 Department of Developmental Biology, Washington University Medical School, Saint Louis, Missouri, United States of America; Inserm U606 and University Paris Diderot, France

## Abstract

In this study using genetic approaches in mouse we demonstrate that the secreted protein Wise plays essential roles in regulating early bone formation through its ability to modulate Wnt signaling via interactions with the Lrp5 co-receptor. In Wise^−/−^ mutant mice we find an increase in the rate of osteoblast proliferation and a transient increase in bone mineral density. This change in proliferation is dependent upon Lrp5, as *Wise;Lrp5* double mutants have normal bone mass. This suggests that Wise serves as a negative modulator of Wnt signaling in active osteoblasts. Wise and the closely related protein Sclerostin (Sost) are expressed in osteoblast cells during temporally distinct early and late phases in a manner consistent with the temporal onset of their respective increased bone density phenotypes. These data suggest that Wise and Sost may have common roles in regulating bone development through their ability to control the balance of Wnt signaling. We find that Wise is also required to potentiate proliferation in chondrocytes, serving as a potential positive modulator of Wnt activity. Our analyses demonstrate that Wise plays a key role in processes that control the number of osteoblasts and chondrocytes during bone homeostasis and provide important insight into mechanisms regulating the Wnt pathway during skeletal development.

## Introduction

Recent studies have shown that canonical Wnt signaling plays multiple roles during skeletal development, including the genesis of chondrocytes, osteoblasts and osteoclasts [1]–[4]. The Wnt pathway is important for the early fate decisions taken by the osteochondro-progenitor cell, where high levels of β-catenin promote the osteoblast lineage and low levels of β-catenin lead to the chondrocyte lineage [1], [3]. Later during skeletal development, Wnt activity is also important for chondrocyte proliferation and for the regulation of bone homeostasis [2], [5]. Bone homeostasis is a finely controlled process whereby a balance is maintained between cells responsible for building bone (osteoblasts) and cells responsible for removing bone (osteoclasts). Wnt signaling in osteoblasts is required for their proliferation and maturation, and for the expression of the osteoclast differentiation antagonist, osteoprotegerin (OPG) [2]. Thus, the Wnt pathway has a series of direct and indirect inputs into the control of both osteoblastogenesis and osteoclastogenesis, and as a consequence maintaining the proper levels of Wnt activity is critical for bone homeostasis.

The large number of extracellular components associated with Wnt signaling in a variety of tissue contexts, makes this pathway highly complex in nature - 19 Wnt ligands, 10 Frizzled receptors, 2 LRP co-receptors, and the 12 soluble inhibitors (sFRP, Dkk, Sost, and Wise). Yet, the complexity of these inputs provides diverse means for tightly controlling the output and balance of Wnt signal transduction. This also makes it a challenge to define the specific cohort of actual modifiers of Wnt signaling employed in any given tissue or context, such as skeletal development. In light of these difficulties, studies examining the role of the Wnt pathway during bone formation and homeostasis have focused on the more conserved intracellular components of this pathway, and taken advantage of the fact that the canonical Wnt pathway converges upon β-catenin for its activity. Due to the critical role of β-catenin in many tissues and developmental processes, genetic studies involving β*-catenin* have depended upon conditional mouse mutants to investigate its role as a component of the Wnt pathway in skeletal development. These analyses have been complicated because results are dependent upon the timing of β*-catenin* inactivation or activation during osteoblastogenesis. For example, two studies demonstrate a role for Wnt signaling during osteoblastogenesis by conditionally modulating β*-catenin* and/or APC, another conserved intracellular Wnt pathway molecule [2], [4]. Both studies concluded that a deletion of β*-catenin* leads to osteoporosis (low bone mass) and activation of β*-catenin* leads to osteopetrosis (high bone mass). However, one of the studies reported that a deletion of β*-catenin* resulted in increased RANK-L and a decrease in osteoblast number [4], while the other found that a loss of β*-catenin* function leads to an increase in osteoclast number, and no change in osteoblast number [2]. It is important to note that both studies concluded that the loss-of-β*-catenin* function resulted in defects in bone resorption rather than bone formation, which contrasts with the bone formation defects observed upon mutation of the mouse *Lrp5* gene [6]. Recessive loss-of-function mutations that map to the Human *LRP5* gene cause osteoporosis pseudoglioma (OPPG) syndrome, which is characterized by low bone mass, while a dominant gain-of-function mutation results in the High Bone Mass (HBM) trait [6]–[12]. Additional amino acid mutations have been mapped to the first propeller region of LRP5 which also leads to HBM disease [13]–[16]. Although there is a suggestion that *Lrp5* can under certain circumstances modulate bone density through serotonin synthesis in the duodenum [17], more recent studies using mice with osteocyte-specific expression of *Lrp5* alleles clearly indicates that Lrp5 functions directly in these cells to effect changes in bone mass via canonical Wnt signaling [18].

The results in these studies suggest that precise or dynamic maintenance of the levels of activity of the Wnt pathway are essential to potentiate its diverse functions in skeletal development and highlight the need to understand the actual molecular mechanisms that modulate this pathway. In this regard, using a functional screen we previously isolated a gene, *Wise*, encoding a novel and highly-conserved secreted protein. *In vitro* assays revealed that Wise binds to the Wnt co-receptors Lrp5 or Lrp6 and in *Xenopus* assays can activate and inhibit Wnt signaling in a context-dependent manner [19]. In addition, Wise (also known as, USAG-1, Ectodin, and SOSTDC1) has also been shown, by us and others, to selectively bind BMP ligands and inhibit their signaling in culture assays [20]–[22]. Hence, Wise posses the biochemical ability to potentially modulate both Wnt and BMP signaling. This is interesting in relation to bone development because Wise shares high homology to Sclerostin (*SOST*) and together they define a novel sub-group of cysteine knot proteins [19], [20], [23]. *SOST*, positionally cloned as the gene causing Sclerosteosis is a regulator of bone density [24]–[26]. Based on its weak similarity to the DAN and CCN family of cystein knot proteins, which themselves bind BMPs, Sclerostin has been postulated to exert its function in regulating bone density via its biochemical ability to bind BMPs and inhibit signaling [25], [27], [28].

However, we and others have shown that both Wise and Sclerostin can function to inhibit Wnt signaling in *in vitro* assays by binding to Lrp5 or Lrp6 [19], [20], [23], [29]. *In vivo* Wise modulates Wnt-dependent tooth number and patterning through *Lrp5* and *Lrp6* in combination with a negative feedback loop between Wnt and SHH signaling [30]. Wise also modulates Wnt signaling in the formation and patterning of skin appendage placodes through Lrp4 [31]. A more recent study reported that Wise (together with Sclerostin) coordinated digit number through Wnt-dependent misregulation of SHH signaling, suggesting a relationship between Wise function and skeletal development [32]. However, no analyses or comments were made detailing any other skeletal defects. Loss-of-function mutations in the human *LRP5* gene result in a recessive low bone mass syndrome (OPPG) while a G171V substitution results in a dominant high bone mass (HBM) trait [10], [11]. This opens the possibility that *Wise* and *Sost* may regulate bone density in a common manner based on their abilities to modulate Wnt signaling.

To address this question we have used genetic analyses in the mouse to investigate the roles of Wise in bone development. Here we report that Wise has multiple functions in regulating developmental aspects of bone formation through its interaction with the Wnt co-receptor Lrp5. Specifically, we show that Wise modulates chondrocyte and osteoblast rates of proliferation in an *Lrp5*-dependent manner. We also find that *Wise* and *Sost* are differentially expressed in osteoblast cells during temporally distinct phases in a manner consistent with the onset of their respective increased bone density phenotypes. Our findings strongly suggest that *Wise* and *Sost* are key modulators of bone development through the ability of their encoded proteins to interact with Lrps and control the balance or levels of Wnt signaling.

## Materials and Methods

### *Wise* Mutant Mouse (129 Sv/EV & C57BL6)

A *neoLacZ* cassette containing stop codons at the 3′ end was inserted into exon1 of *Wise* at the SmaI site in a genomic fragment isolated from a 129 Sv/EV mouse BAC. The modified BAC was then used for homologous recombination after electroporation into either 129 Sv/EV or C57BL6 mouse ES cells. Specific integrants were selected from Southern analysis using a 3′ probe within exon 2 of *Wise*. EcoRI digests yielded either a 6.8 Kb fragment associated with homologous recombination, or a 9 Kb fragment associated with a random integration event. The mice were genotyped using the following primers (5′-3′ orientation); wildtype forward primer (MA3) “*GCAAGGGGGAAAGAATAAGCG*”; wildtype reverse primer (MB33) “*GATCCAGTCCAGTGCTACTG*”; and *Wise* mutant reverse primer (BG3C2) “*CATCTGCCAGTTTGAGGGGA*”. The resulting PCR products are a wildtype band at 250 bp and *Wise* mutant band at 401 bp. Characterization of mutant animals revealed that for unknown reasons the LacZ gene in the targeting site is not expressed.

### Immunochemistry (IHC)

Mouse femurs were harvested from post natal day 0 (P0), fixed in 4% paraformaldhyde and processed for standard paraffin sectioning. H&E was carried out using standard histological techniques. IHC was preformed using PBS (with or without Triton) and 10% goat serum [16]. The primary antibody was a custom made murine Wise anti-peptide rabbit polyclonal antibody (1∶200) [20]. Five male animals were used for each genotype and stage unless stated otherwise. Control samples that included no primary antibody and pre-immune sera instead of primary antibody were used to determine specificity of all antibodies [16].

### Bone Density

Bone densitometry was measured on whole body using a PIXImus mouse densitometer (GE medical systems). Bone mineral and body composition were measured using Dual X-ray absorptiometry (DEXA). The whole mouse, including hands and feet, were carefully placed within the PIXImus data square. A minimum of five animals from each sex, mouse strain and genotype were used per stage unless stated otherwise. Two tailed student T-Test statistics was used with a confidence interval of p<0.05 to identify level of significance.

### TRAP and ALP

Alkaline phosphatase (ALP) staining was preformed on cryosectioned E14.5 mouse femurs. ALP staining was carried out for 15 minutes at Room Temperature (RT) in 100 mM Tris-maleate (pH 9,2), Naphthol As-MX phosphate and Fast red TR. For ALP on Mouse femurs at P0, plastic sections were used instead of cryosections, and NBT/BCIP instead of fast red. TRAP (tartrate resistant acid phosphatase) staining was done on paraffin sections from E14.5 mouse femurs as per manufactured protocol for Sigma acid phosphatase kit (cat. 181A). A minimum of five male animals were used from each genotype and stage unless stated otherwise.

### Bone *In Situ* Hybridization (ISH)

E14.5 mouse femurs were fixed in 4% paraformaldhyde for 2 overnights at 4°C. 5 µm cryosections were dried for 2 hours to overnight at room temperature. Slides were sequentially rinsed in water, PBS an 2XSSC. Hybridization was at 65°C overnight in a humidified chamber using 1 µg/ml dig-labeled probe. Post-hybridization washes at 60°C were for 2×10 minutes in 50% formamide, 1XSSC, 0.1% tween-20, followed by 2×10 minutes in MABT and 30minutes with blocking buffer (20%goat serum, 20%BBR, 60% MABT). Anti-dig AP was added at 1∶2000 and incubated overnight at room temp. Color was revealed using NTMT with NBT/BCIP and stopped with PBST. RNA probes *ColII* and *ColX* were from Gerard Karsenty, *Sox9* from Paul Sharpe and *Wise* and *Sost* probes were generated from the 3′UTR of a mouse BAC containing genomic sequences from *Wise* and *Sost* that were subcloned into pBluescript. A minimum of five animals were used from each genotype and stage unless stated otherwise.

### Skeletal Stain

Mouse P0 whole bodies were fixed in 4% paraformaldhyde for 2 overnights at 4°C, then into 95% ETOH for 7 days, and acetone for 1 day. The mice were then stained in standard alizarin red/alcian blue solution at 37°C for 3 days and rinsed in water. Then cleared using 1%KOH for 1 day, and placed in a series of increasing Glycerin (20%- 3 days, 50%- 4 days, 80%- 2 days)/1% KOH until a final concentration of 100% Glycerin for 2 days is achieved. A minimum of five animals were used for each genotype and stage unless stated otherwise.

### BrdU Proliferation Assay

BrdU (Sigma B-9285) was prepared in cold PBS at a concentration of 10^−4^M. The BrdU was injected into the nape of the neck of newborn pups [2]. The pups were then terminated after 2 hours. The tissue of interest was then dissected out and fixed in 4% paraformaldhyde overnight at 4°C, then washed in cold PBS. The samples were ETOH dehydrated, paraffin embedded and sectioned at 5 um. Antigen retrieval used a Citrate buffer and Biogenex EZ retriever microwave. Immunostaining was automated using Biogenex and antibodies from Amersham. A minimum of five animals were used for each genotype and stage unless stated otherwise.

### Calcein (Bone Formation Rate) Assay

Calcein (Sigma C-0875) was made up to a concentration of 2.5 mg/ml and injected on days 1 and 4, and terminated on day 6 [2]. The tissue was then dissected and fixed in 4% paraformaldhyde overnight at 4°C. Paraffin embedded and processed at 7 um. A minimum of five animals were used for each genotype and stage unless stated otherwise.

### Toluidine Blue Staining and von Kossa Stain

1 g Toluidine Blue (EM, CAS 92-31-9) was diluted in distillated water then filtered [2]. Slides were incubated for 5–10 min then rinsed in distillated water, dehydrated and cover-slipped. For von Kossa staining, samples were fixed in 4% paraformaldhyde overnight at 4°C, and then paraffin embedded and processed at 5 um. A 5% silver nitrate was used to stain slides in direct sunlight for 10–20 minutes. The slides were then rinsed in water and then the reaction stopped by the addition of 5% sodium thiosulphate for 2–3 minutes. A minimum of five male animals were used for each genotype and stage unless stated otherwise.

### Histomorphometry

Osteomeasure software was used to accurately quantitate the number of Toluidine Blue stained active osteoblasts and TRAP positive osteoclasts from 1.5 month, 2.5 month or 4 month mouse tibia long bone [2]. Six comparable slides containing two sections within the middle of the tibia were used for each sample. A distance of 800 um was analyzed from the growth plate into the primary spongiosa. The bone formation rate was also quantified using osteomeasure setting the time of injection to 4 days for the metaphysis of the tibia long bone from a 1.5 month or 2.5 month mouse. The trabecular bone volume was assessed in Von Kossa stained using both mouse tibia long bones and L5 vertebrae from tissues at 1.5 month, 2.5 month, and 4 month. Samples consisted of a minimum of five male animals per genotype per stage unless stated otherwise.

### Animal Care and Usage

This study was carried out in strict accordance with the recommendations in the Guide for the Care and Use of Laboratory Animals of the National Institutes of Health. The protocols were approved by the Institutional Animal Care and Use Committees of the Stowers Institute for Medical Research (RK Protocol ID: 2013-0110) and Washington University School of Medicine (SS Protocol ID: 20120279). Animals were euthanized by carbon dioxide consistent with the recommendations of the Panel on Euthanasia of the American Veterinary Medical Association, and all efforts were made to minimize suffering.

## Results

**Figure 1:**
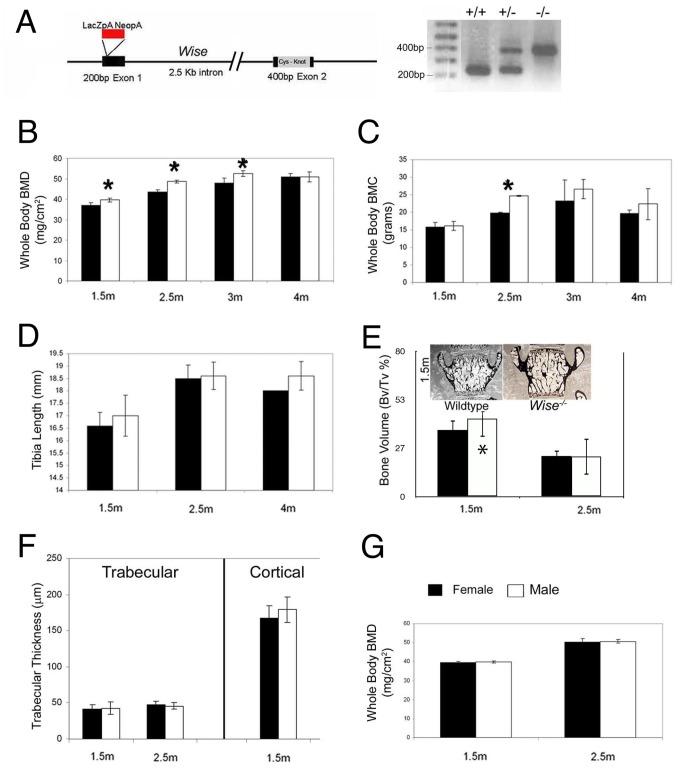
Wise^−/−^ Mutant Animals Display a High Bone Mass Phenotype. (A) Genomic structure of *Wise*. The *Wise^−/−^* mutant was created by insertion of a *neoLacZ* cassette (red) into exon 1 at the SmaI site. Panel on the left illustrates *Wise* PCR based genotyping; genomic fragments are generated for a wildtype band at 250 bp and a mutant band at 401 bp. (B) Whole body bone mineral density (BMD) measurements of the *Wise* ^−/−^ mutant animals. *Wise* ^−/−^ animals have a significant (p<0.05, asterisks) increase (7.5–11.6%) in bone density from between 1.5 to 3.0 months. At 4 months bone density normalized in *Wise* ^−/−^ mutant animal. Black bars represent wildtype, and white bars *Wise* ^−/−^ mutants. (wildtype/mutant: N = 6/6 1.5 m; N = 3,3 2.5 m; N = 12,3 3 m; N = 8,2 4 m). (C) Whole body mineral content (BMC) was increased significantly only at 2.5 months. No significant changes in bone mineral content were observed at 1.5 months or at 3 months of age. Thus increases in bone mass seen at 1.5 & 3 m are not due to significant increases in whole body bone mineral content. (wildtype/mutant: N = 6/6 1.5 m; N = 3/3 2.5 m; N = 12/3 3 m; N = 8/2 4 m). (D) To address gross chondrocyte changes in the growth plate we measured the length of the tibia by X-ray. When compared to wildtype, the *Wise^−/−^* mutant did not display any significant difference in the length of the tibia. (wildtype/mutant: N = 5/4 1.5 m; N = 6/5 2.5 m; N = 3/3 4 m). (E) Histomorphometry of trabecular bone volume tabulated from von Kossa stained L5 vertebrae (equal results were obtained from Von Kossa stained long bones). *Wise* ^−/−^ mutants (white bars) display a significant 1.3 to 1.5 fold increase over wildtype (black bars) in trabecular bone volume at 1.5 month and 2.5 month of age. Increases of trabecular bone volume in *Wise* ^−/−^ animals is normalized to wildtype levels. (wildtype/mutant: N = 6/6 1.5 m; N = 5,3 2.5 m). (F) Histomorphometry of trabecular and cortical thickness of long bones. *Wise* ^−/−^ mutant mice do not display a significant change, over wildtype, in either trabecular or cortical thickness. (wildtype/mutant: trabecular N = 6/6, cortical N = 6/6 1.5 m; trabecular N = 5/3 2.5 m). (G) Comparison of bone mineral density between males and females *Wise^−/−^* mutants. There is no significant difference seen in the *Wise* ^−/−^ mutants between the sexes at either 1.5 month or at 2.5 months of age. (female/male: N = 3/3 1.5 m; N = 4/3 2.5 m). Statistically significant (Student T-Tests, two tailed, p<0.05) values are demarked by an asterisks.

In this study, to address the functional roles of *Wise in vivo* and examine its relationship to *Lrp5*, we have generated a targeted null mutation in the mouse gene by insertion of a selection cassette following the signal sequence in exon 1 (Fig. 1A). The *lacZ* gene inserted into the locus is not expressed. The effects of this targeted mutation have been analyzed in both the 129 Sv/EV and C57BL6 background strains. The comparison of the Wise mutants within each strain, has revealed that in both strains, heterozygous *Wise^+/−^* mice are viable and fertile, and display no obvious phenotypes. Homozygous *Wise^−/−^* mice are also viable and fertile, however in the 129 Sv/EV strain litter sizes are often reduced and there is a frequent increase in the number of resorptions. Postnatal homozygous mutant animals display a number of distinct phenotypes [25] [26], including changes in bone density which are described in this study.

### *Wise* Mutant Mice Display Increased Whole Body Bone Mineral Density

*In vitro* assays suggest that Wise and Sclerostin have the potential to inhibit Wnt signaling via a Lrp1, Lrp4, Lrp5 or Lrp6-dependent mechanism, [19], [20], [23], [29]. This potential, in combination with bone density phenotypes in human *SOST* and *LRP5* mutations, led us to examine whether *Wise* participates in regulating bone mass. We found a significant increase (p = 0.0017) in C57BL6 *Wise−/−* whole body BMD over C57BL6 wildtype (n = 99), a difference that was more pronounced than we observed when comparing 129 Sv/EV *Wise−/−* and wildtype 129 Sv/EV (n = 55) animals (Data not shown, numbers reflect total animals between control and test group). Because of the more severe phenotype in the C57BL6 strain we chose to focus on this strain for a more detailed analysis. C57BL6 *Wise^−/−^* mutant mice have an increased whole body bone mineral density (BMD) (7.5–11.6%) during the first 1.5 to 3 months of postnatal development (Fig. 1B). Interestingly, by 4 months of postnatal development the increase in bone density normalizes in the *Wise^−/−^* animals and is restored to strain wildtype levels. This correlates with Beamer et al (1996) who reported that bone density in various mice strains undergoes an increase in bone deposition from 2 months (and possibly earlier) to 4 months, after which bone deposition appears to be at equilibrium with bone resorption [33]. Thus Wise is acting as an early (developmental) regulator of bone deposition. Coincident with the initial increase in BMD observed using DEXA in *Wise^−/−^* mutant animals there is a significant (p<0.05) increase in trabecular bone volume at 1.5 and 2.5 months (Fig. 1E; asterisk) as observed using histomorphometry. The increase in BMD in *Wise^−/−^* animals is not due to increases in bone mineral content (BMC), as BMC values appear to be only significantly increased in the *Wise^−/−^* mutants at 2.5 months (Fig. 1C) observed using DEXA. There is also no difference in tibia length (Fig. 1D), in trabecular or cortical thickness (Fig. 1F) as observed using histomorphometry, or whole body bone mineral density between males and females (Fig. 1G) observed using DEXA. Hence in *Wise^−/−^* animals there is a significant transient increase in early bone density.

### Genetic Correction of *Wise* with *LRP5* during Mineralization

**Figure 2:**
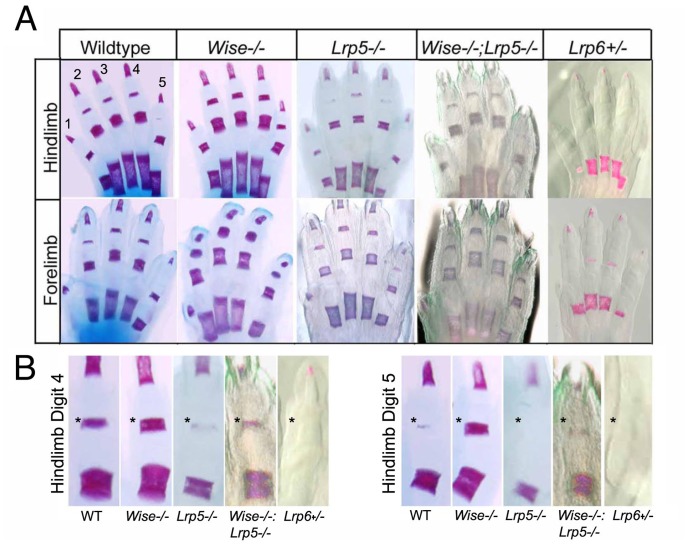
Genetic Correction of *Wise* with *Lrp5* during Ossification. (A) Skeletal stain at P0 showing hindlimb and forelimb phalange development in the wildtype, *Wise^−/−^*, *Lrp5^−/−^*, *Wise^−/−^:Lrp5^−/−^* and *Lrp6^+/−^* strains. (B) Asterisks (*) highlight an increase in the ossification centers of digits 4 and 5 of the hindlimb in *Wise^−/−^* mutants compared to the other strains. This *Wise^−/−^* mutant phenotype in digits is partially rescued in an *Lrp5^−/−^* strain showing an epistatic relationship between *Wise* and *Lrp5*. n = at least 5.

Consistent with changes in bone density, in C57BL6 *Wise^−/−^* mutant animals, we observe an increased mineralization of phalanges in both the fore and hindlimbs, which is clearly evident in digits 4 and 5 (high power panels, asterisks, Fig. 2). This phenotype is fully penetrant in both strains. This increased digit mineralization phenotype is the opposite to that observed in both *Lrp5^−/−^* and *Lrp6^+/−^* mutant animals, which display a reduced mineralization of the phalanges ([6], [34] and Fig. 2). To determine if the 129 Sv/EV *Wise^−/−^* digit phenotype is also dependent upon *Lrp5*, we generated *Wise^−/−^;Lrp5^−/−^* double mutant animals and compared them with phenotypes in the single *Lrp5^−/−^* and 129 Sv/EV *Wise^−/−^* mutants. Analysis of digits in *Lrp5^−/−^* mutant animals confirmed that there is a reduced mineralization of phalanges at P0 (Fig. 2A and 2B) [6], although we find the phenotype variable and not fully penetrant. In *Wise^−/−^;Lrp5^−/−^* double mutant animals, the increased mineralization seen in digits of 129 Sv/EV *Wise^−/−^* mutants is restored to near wildtype levels or those seen in the single *Lrp5^−/−^* mutant animals (Fig. 2A and 2B). This demonstrates a genetic interaction between *Wise* and *Lrp5* in the digits and indicates that *Lrp5* functions down-stream of *Wise* in digit mineralization. This is also in agreement with the model that Wise can function as a modulator of Wnt signaling via its interactions with the Lrp5 or Lrp6 co-receptors. The *Wise* digit phenotype may also be dependent upon *Lrp6*, as we have found that *Lrp6^+/−^* animals have an even more severe reduction in mineralization of the phalanges (Fig. 2), similar to that seen in the *ringelschwanz Lrp6^R886W/R886W^* homozygous animals. Our results illustrate that both *Lrp5* and *Lrp6* play roles in regulating digit ossification (Fig. 2). This is also consistent with published findings that reveal dose-dependent phenotypes and variable penetrance in double *Lrp5/Lrp6* mutant combinations, indicating an epistatic relationship between these two genes in a variety of tissue contexts [35], [36].

### Temporally Dynamic Requirement for *Wise* in Regulating Bone Formation

**Figure 3:**
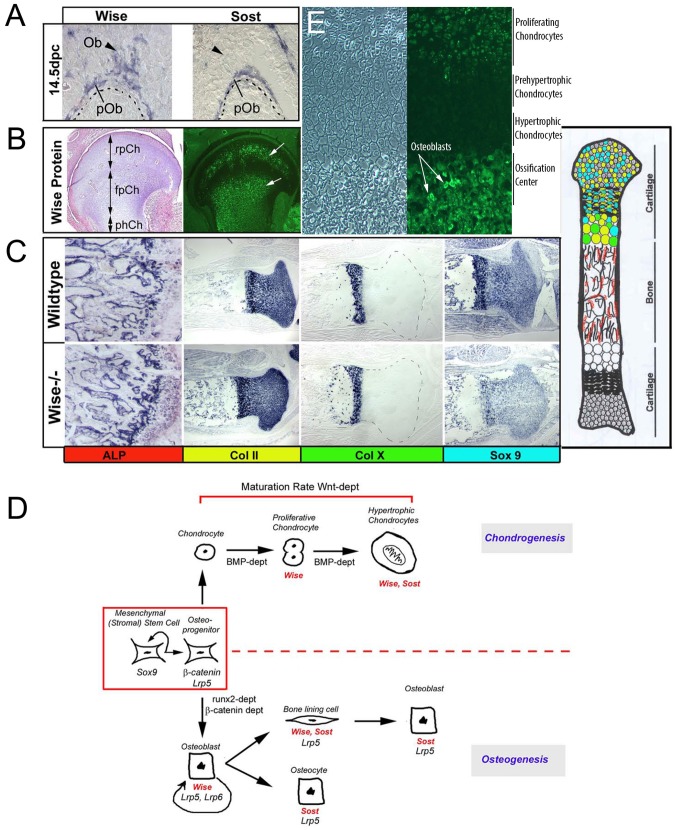
Temporally Dynamic Requirement for *Wise* during Bone Mass Regulation. (A) At E14.5 *Wise* is expressed in the mesenchymal pre-osteoblasts and periosteum while *Sost* is only expressed in the pre-osteoblasts of the ulna. pOb, pre-osteoblasts. (B) Avian wise protein localization in a prenatal embryonic Day 18 tibia (HH stage 44). Wise protein (FITC fluorescence coupled to the Wise primary peptide antibody) is found in the round and a subset of flat proliferative chondrocytes (arrows) as well as pre-and hypertrophic chondrocytes. pCh; proliferating chondroctyes, rpCh; round proliferative chondrocytes, fpCh; flat proliferative chondrocytes, phCh, prehypertrophic chondrocytes, hCh; hypertrophic chondrocytes. (C) Comparing expression of bone specific markers between wildtype and *Wise* ^−/−^ animals at P0. ALP appears to have increased staining in the *Wise* ^−/−^ growth plate and primary spongiosa. *ColII, ColX* and *Sox9* show no significant change in expression pattern in the *Wise^−/−^* animals, when compared to wildtype. Normal patterns of ALP (red), *Col II* (yellow), *Col X* (green), and *Sox9* (blue) are shown in the bone schematic on the far left. (D) Model integrating *Sost* and *Wise* expression during development of both chondrocytes and osteoblasts lineages. In this scheme Wise acts in early osteoblasts while Sost functions later in mature osteoblasts and osteocytes. In chondrocytes, Wise and Sost appear to act both in the hypertrophic chondrocyte. n = at least 5 in most cases. (E) In murine, at Prenatal embryonic day 14, Wise protein is also localized to a subset of proliferating chondrocytes and active osteoblasts on the surface of the trabeculae in the ossification center (arrows) but not in the hypertrophic chondrocytes.

To investigate the cellular mechanisms that underlie the increase in bone mass and its transient nature, we have examined *Wise* expression during long bone development and used bone specific markers to characterize changes in the C57BL6 *Wise^−/−^* mutants. At E14.5 *Wise* mRNA is expressed in the mesenchymal pre-osteoblasts (pOb) and periosteum, based on alkaline phosphatase (ALP) staining as a marker for late osteoblast matrix deposition (Fig. 3A ). At E14 in mice Wise protein is excluded from the hypertrophic chondrocytes but is found in active osteoblasts lining the trabeculae and the proliferating chondrocytes (Fig. 3B & E) conversly in avian at a slightly later stage pre-natally (Hamburger and Hamilton Stage 44 ca. 18 days) Wise avian protein can be detected in a subset of round proliferative chondrocytes, flat proliferative chondrocytes, and hypertrophic chondrocytes, but at 4 months of age it is no longer expressed in the active osteoblast lineage (data not shown). Hence, *Wise* is expressed by mesenchymal preosteoblasts during the early period of bone formation; consistent with our results showing increased bone density in the *Wise^−/−^* mutants between 1.5 and 3.0 months of age (Fig. 1B). At P0, *Wise^−/−^* mutant animals have increased ALP staining when compared to wildtype animals consistent with increased BMD (Fig. 3C). Chondrocyte markers *ColII, ColX*, and *Sox9* staining show that the different chondrocyte layers, which are present in wildtype, are also present in the *Wise^−/−^* mutants (Fig. 3C). However, the *Wise^−/−^* has punctate staining of *ColX* within the primary spongiosa, which is not present in wildtype mice of similar strains.

The restoration of normal bone density after 4 months in the *Wise^−/−^* mutant animals correlates with the temporal loss of *Wise* expression in the active osteoblast lineage. Hence, functional compensation by other proteins might account for the transient nature of the *Wise* BMD phenotype. In this regard, Wise is closely related to the human protein Sclerostin (SOST), thus we have also examined the expression of mouse *Sost* during bone development. At E14.5 *Sost* is expressed in pre-osteoblast cells but not in active osteoblasts (Fig. 3A). Conversely, at 4 months of age *Sost* is expressed in both osteoblasts and osteocytes, while *Wise* expression in the osteoblast lineage is absent (data not shown). Hence, *Wise* and *Sost* are differentially expressed in osteoblast cells during temporally distinct phases, whereby *Wise* is expressed early and *Sost* later. Fig. 3D summarizes the timing of *Wise* and *Sost* expression during chondrocyte and osteoblast differentiation. These expression patterns are consistent with the late onset of increased bone density in human Sclerosteosis patients [37]. Hence, there is a strong correlation for both *Wise* and *Sost* between the timing of their osteoblast gene expression and the onset of their respective increased bone density phenotypes.

### *Wise* Regulates Chondrocyte and Osteoblast Proliferation

**Figure 4:**
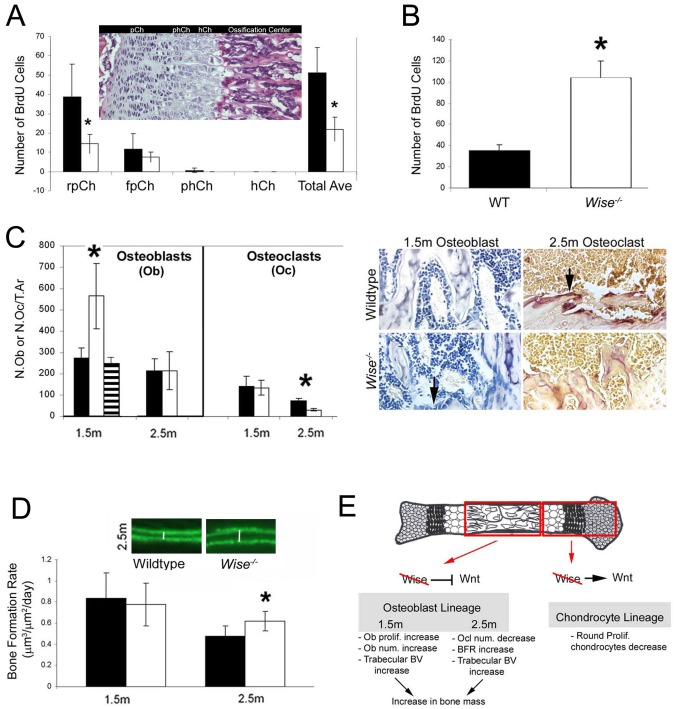
Characterization of the Osteoblast and Chondrocyte Phenotype in Wise^−/−^ Mutants. (A) Chondrocyte proliferation as detected using BrdU. P4 *Wise* ^−/−^ mutants display a significant (asterisk) 63% decrease in proliferation of the round proliferative chondrocytes (rpCh), whereas no significant change in the flat proliferative (fpCh), prehypertrophic (phCh), and hypertrophic chondrocytes (hCh). Black bars depict wildtype, white bars depict *Wise* ^−/−^. Insert shows the location of the different chondrocyte types within the long bone femur of a mouse. pCh; proliferating chondrocytes. (N = 4 per group). (B) Active osteoblast cell proliferation as detected using BrdU in a 1.5 month mouse. *Wise* ^−/−^ mutants display a significant (asterisk) 2.9 fold increase in active osteoblast proliferation of long bones at 1.5 months. (N = 4 per group). (C) Active osteoblast/osteoclast numbers counted from Toluidine Blue or Trap stained sections (Toluidine blue/Trap stained panel adjacent to chart) are quantified in Chart. A 2 fold significant increase (asterisk) in active osteoblasts is seen in 1.5 month old *Wise* ^−/−^ mutant long bones (arrow, blue columnar cells adjacent to trabeculae in adjacent Toluidine Blue panel). This increase in active osteoblast numbers is dependent on the presence of Lrp5 as shown by the Chart hatched bar at 1.5 month (*Wise^−/−^;Lrp5^−/−^*). Osteoclast numbers (adjacent Trap stained panel (red), arrow) counted from TRAP stained sections reveal a significant 58% decrease in numbers at 2.5 months of age in a *Wise* ^−/−^ mutant. (wildtype/mutant: osteoblasts N = 6/6 1.5 m, N = 5/3 2.5 m; osteoclasts N = 6/6 1.5 m, N = 5/3 2.5 m). (D) Bone formation rate as assayed from calcein double labeling. A significant (asterisk) 1.3 fold increase in bone formation rate is evident in *Wise* ^−/−^ mutant mice at 2.5 months of age. (N = 3 per group). (E) Summary of Wise Function. Wise functions as both a stimulator and an inhibitor of Wnt signaling during skeletal development. In the chondrocytes, Wise acts as a positive stimulator of Wnt signaling, whereas in the osteoblast, Wise functions as a Wnt antagonist. A loss of Wise function leads to an increase in bone mass at 1.5 to 2.5 months of age. The increase in bone mass at 1.5 months of age is attributed to an increase in osteoblast Wnt signaling and hence osteoblast proliferation, resulting increase in bone volume. Whereas, the increase in bone mass seen at 2.5 months is due to a decrease in osteoclast number possibly from increased osteoblast Wnt signaling that results in an increase in OPG levels, and resulting decrease in resorption. This potential decrease in resorption could result in the increased bone mass at 2.5 months. However at 2.5 months the bone formation rate is also increased and this would lead to the increase bone mass and increased trabecular bone volume.

The mineralization phenotype observed in C57BL6 *Wise^−/−^* mutants and its genetic interaction with *Lrp5^−/−^* mutants imply that Wnt signaling has been altered in chondrocytes and osteoblast cells, therefore we analyzed these cell types from C57BL6 mice in more detail (Fig. 4). Using BrdU labeling, we found that the proliferation of the round proliferative chondrocytes is significantly (p<0.05) decreased by 62% at P0, in the absence of *Wise* (Fig. 4A). This is consistent with the idea that Wise acts as a positive stimulator of Wnt signaling within the chondrocyte (Fig. 4E). In contrast, active osteoblast proliferation is significantly increased by 2.9 fold at 1.5 months post-natally in the absence of *Wise* (Fig. 4B), in agreement with its role as a Wnt antagonist in active osteoblasts (Fig. 4E). The increase in trabecular osteoblast proliferation at 1.5 months assayed by BrdU incorporation is consistent with a significant increase in the number of active osteoblasts as quantified using Toluidine Blue staining (Fig. 4C). The increase in trabecular osteoblast number and proliferation at 1.5 months further correlates with a significant increase in trabecular bone volume (Fig. 1E). To further investigate the potential link between Wise and Wnt signaling in this context we generated *Wise^−/−^*:*Lrp5^−/−^* mutants. We find that the increase in active trabecular osteoblast numbers in *Wise^−/−^* animals at 1.5 months is also dependent upon the presence of *Lrp5*, as in double mutant animals the numbers of active osteoblasts normalize to wild type levels (Fig. 4C, hatched bar).

At 2.5 months post-natally, loss of *Wise* leads to a significant decrease (58%) in the number of trabecular osteoclast cells (Fig. 4C). This may be a consequence of an earlier increase in Wnt signaling, osteoblasts and OPG levels, which negatively regulates osteoclast differentiation [2]. At 2.5 months we also observe a significant increase in trabecular bone volume (Fig. 1E) and 38% increase in osteoblast bone formation rate (Fig. 4D). In summary, our results suggests that *Wise* functions to regulate bone development in multiple ways by modulating Wnt signaling in an Lrp-dependent manner that ultimately regulates the number of chondrocytes, active trabecular osteoblasts and osteoclasts.

## Discussion

In this study we demonstrate that Wise plays a fundamental role in regulating early bone deposition through its ability to negatively modulate Wnt signaling via interactions with the Lrp5 co-receptor. *Wise* ^−/−^ mutants display a significant (10%) increase in bone mass at 1.5 months, due to increased proliferation of active osteoblasts. This increase is dependent upon functional *Lrp5*, as *Wise; Lrp5* double mutants have normal bone mass, implying this function is mediated by activation of Wnt signaling in these cells. Furthermore, *Wise* has additional roles in bone formation outside of osteoblast proliferation, as we found that it is required to potentiate proliferation in chondrocytes as well. These findings suggest that *Wise* has a role in fine-tuning Wnt signaling during skeletal development through combined inputs on the control of both osteoblastogenesis and chondrogenesis. Therefore, this work provides important insight into mechanisms regulating the Wnt pathway during the process of bone homeostasis.

### Canonical Wnt Signaling and Bone Formation

Our analysis of *Wise* mutants, along with published studies of *Lrp5* and *Axin2* mutants [6], [38], reveal that these mutants all display changes (an increase or decrease) in the number of actively proliferating osteoblasts and possess corresponding bone formation phenotypes. These analyses underscore the importance of Wnt signaling in mechanisms regulating bone homeostasis via osteoblasts. Therefore, it is interesting that a loss of *β-catenin* does not mimic the phenotypes seen in these three mutants [2], [4], [39]. Mutation of β-catenin affects bone resorption, as opposed to bone formation, two very distinct aspects of bone homeostasis.

The absence of an effect on osteoblast proliferation in *β-catenin* mutants is surprising because of the pivotal role β-catenin plays in transducing canonical Wnt signaling. Modulation of Lrp5 or soluble Wnt inhibitors like Wise may function to alter one of the main inputs of Wnt signaling and control levels of output, but there could be other inputs, for example mediated through Lrp6. In contrast, because β-*catenin* plays such a critical role in transducing canonical Wnt signaling, its absence would be expected to have a more drastic affect on the relative levels of activity. So how does one explain that the loss of β-*catenin* does not lead to changes in osteoblast number as in *Wise*, *Lrp5* and *Axin2* mutant mice? It could be that the conditional β-*catenin* mouse mutants were generated with drivers activated too late to give a bone formation phenotype, and instead display only a bone resorption phenotype. To resolve this issue, Rhodda and McMahon (2006) examined a later inactivation of β-*catenin* and have shown that this does indeed lead to an expansion of the osteoblast cell population [40]. Thus it appears that the Wnt pathway plays multiple roles during osteoblast specification and also osteoblast population expansion.

### Temporal Dynamics and Common Functions for *Wise* and Sclerostin in Bone

This study demonstrates that in the bone Wise plays a key *in vivo* role in modulating Wnt activity in an *Lrp5*-dependent manner. The transient increase in bone density observed in *Wise* mutants correlates with the expression of this gene in early active osteoblasts lining the trabeculae. The increase in osteoblast number and decrease in osteoclast number is reminiscent of uncoupling bone resorption from bone formation [2]. The inverse is seen for osteoblast numbers in a *β-catenin* [41] or an *Lrp5* [6] knockout which leads to a loss of osteoblast proliferation. Wnt signalling has been reported to effect osteoblast proliferation, thus one could hypothesize that a loss of Wnt signaling (as in the *β-catenin* or *Lrp5* null mutants) results in a loss of proliferation whereas a gain in Wnt signaling (as in *Wise* mutants) would lead to increased osteoblast proliferation.

Since we find that *Wise* is not expressed in osteoblasts or osteocytes four months after birth, other factors or pathways acting in a parallel or overlapping fashion may compensate for the loss of Wise activity. *Wise* and *Sost* are duplicated and diverged genes that encode a sub-family of cystein knot proteins with weak similarity to the DAN and CCN sub-families. Our expression studies reveal that *Sost* is not expressed in early active osteoblasts at the same time as *Wise*, but it is expressed in later stages when *Wise* is absent. In view of the sequence similarity between Wise and Sclerostin, the differences in timing of expression and the evidence showing that mutations of *SOST* in human and mouse result in high bone density phenotypes, *Sost* is a likely candidate to compensate for the loss of *Wise* in later stages of bone formation. Furthermore, although loss of *Wise* function results in a 62% reduction in hypertrophic chondrocyte proliferation, we interestingly observed no differences in long bone length or body size. While one explanation for this finding could be the lack of expression of *Wise* by hypertrophic chondrocytes in mice, another more plausible explanation could be because Sclerostin is still present in these animals and has the ability to functionally compensate for the lack of Wise. Using the same *in vitro* binding and Xenopus assays employed to investigate the relationship between Wise and Wnt signaling [19], we and others have found that Sost is also able to inhibit various Wnt signaling ligands (Wnt1 and Wnt3a) through interactions with Lrp1, Lrp4, Lrp5 or Lrp6 [20], [23], [42]. Therefore, Sost, like Wise, has the potential to function as a negative regulator of bone deposition by modulating Wnt signaling at later periods of osteoblast maturation, when it is expressed. This suggests a model whereby, Wise and Sost have common roles in regulation of bone mass through the control of Wnt signaling and osteoblast proliferation, although they act at different developmental stages. This is consistent with the late onset of bone density phenotypes observed in human mutations of *SOST*, presumably masked by the earlier transient expression of *Wise*.

### Unique Roles for *Wise* in Wnt Signaling and Bone

The characterization of changes in the rate of both osteoblast and chondrocyte proliferation clearly revealed that Wise functions in a different manner in these two cell types. In the osteoblast, Wise normally serves to reduce or inhibit Wnt signaling, whereas as in chondrocytes Wise may be acting to stimulate Wnt signaling. These opposing roles in Wnt signaling are consistent with our previous analyses in Xenopus embryos, which suggested that Wise has the potential to activate or inhibit Wnt signaling in a context-dependent manner [19]. This differs from other cystein knot family proteins, such as CTGF, Cyr61 and Sost, which also bind Lrp1, Lrp5 or Lrp6 but only appear to be capable of inhibiting Wnt signaling in Xenopus assays [20], [43], [44]. Hence, investigating the mechanism by which Wise exerts this opposing influence on Wnt signaling is important for understanding the control of proliferation in osteoblasts versus chondrocytes. The basis for this context-dependent shift in modulatory potential unique to Wise is unknown. However, it could arise due to different sites of interaction within the second YWTD propeller domain of the LRPs and the cystein knot proteins or from differences in the combination of specific LRP and frizzled co-receptors, Wnt ligands and/or other co-factors (e.g. Dkk), all of which might alter the conformation and stability of the signaling complex on the membrane. Since Wise and Sost, are a sub-set of a large family of cystein knot proteins, other members of this family (slits, mucins, gremlin) may have previously unappreciated inputs into modulation of Wnt signaling in bone formation, and could also influence the specificity of protein interactions and signaling activity.

In summary, *Wise* functions in bone development. Our genetic analyses with *Wise* and *Lrp5* mutants demonstrate that the biochemical interactions between Wise, Lrps and Wnt signaling are functionally important *in vivo* in multiple aspects of bone development. The genetic interaction with *Lrp5* and increased bone density phenotypes we observe in *Wise^−/−^* mutant animals highlights the key role that *Lrps* and Wnt signaling play in bone homeostasis and illustrate the importance of extracellular modifiers, such as Wise, in regulating this pathway.
