# Correction: *Wise* Regulates Bone Deposition through Genetic Interactions with *Lrp5*

**DOI:** 10.1371/journal.pone.0104467

**Published:** 2014-07-29

**Authors:** The PLOS ONE Staff

In the Materials and Methods section, under the subheading “Animal Care and Usage”, the protocol number approved by the Institutional Animal Care and Use Committees of the Stowers Institute for Medical Research is incorrect. The correct protocol number is RK Protocol ID: 2013-0114.
